# Effect of Growth Mindset on Mathematics Achievement Among Chinese Junior High School Students: The Mediating Roles of Academic Buoyancy and Adaptability

**DOI:** 10.3390/bs14121134

**Published:** 2024-11-26

**Authors:** Mudan Chen, Ida Ah Chee Mok, Yiming Cao, Tommy Tanu Wijaya, Yimin Ning

**Affiliations:** 1 School of Mathematical Sciences, Beijing Normal University, Beijing 100875, China; caoym@bnu.edu.cn (Y.C.); 202139130001@mail.bnu.edu.cn (T.T.W.); 2 Faculty of Arts and Sciences, Beijing Normal University, Zhuhai 519085, China; 3 Faculty of Education, The Chinese University of Hong Kong, Hong Kong, China; ahcheeidamok@cuhk.edu.hk; 4 School of Mathematical Sciences, East China Normal University, Shanghai 200241, China; 52274800001@stu.ecnu.edu.cn

**Keywords:** growth mindset, academic buoyancy, adaptability, mathematics achievement

## Abstract

A growth mindset is crucial for students’ academic development. Many studies have demonstrated the influence of a growth mindset on mathematics achievement, but the mediating mechanisms underlying this relationship still warrant further exploration. This study investigates the mediating roles of students’ academic buoyancy and adaptability in the relationship between the student growth mindset and mathematics achievement within the Chinese cultural context. The sample included 1164 junior high school students (49.4% females). Using structural equation modeling, the results showed that, after controlling for gender, school type, and family socioeconomic status, the student growth mindset was positively related to mathematics achievement. Furthermore, the student growth mindset was significantly associated with the student academic buoyancy, cognitive-behavioral adaptability, and affective adaptability, but only cognitive-behavioral adaptability further mediated the relationship between the student growth mindset and mathematics achievement. The multi-group analysis demonstrated that the model exhibited invariance across the genders, school types, and SES levels, indicating that the associations were applicable to both boys and girls, to both boarding and day students, and to students from low-, middle-, and high-SES backgrounds. We discuss the findings by considering the Chinese cultural characteristics and provide insights that may help in the development of interventions to improve students’ mathematics performance.

## 1. Introduction

Rooted in the social-cognitive model of achievement motivation, the concept of the mindset refers to learners’ implicit beliefs or theories about their characteristics [1]. Researchers have argued that a growth mindset of intelligence (i.e., the belief that intelligence is malleable) plays an important role in students’ academic learning [2,3,4]. According to the implicit theories model put forward by Dweck et al. [5], individuals’ implicit beliefs about human attributes may influence their inferences, reactions, and judgments and as a result, lead to different outcomes. In the context of academic life, this model suggests that implicit theories of intelligence determine students’ approach to learning and the goals which they pursue, and ultimately influence their achievement through the mediation of effort expenditure and persistence [6]. Students typically encounter a range of novel circumstances and everyday challenges. The way that students navigate these changes and adversities significantly affects their academic futures. While many experience declines in school engagement and increases in academic anxiety, there are also those who successfully overcome these challenges and even thrive. These phenomena have captured the attention of researchers, particularly those specializing in positive psychology, who are motivated to investigate the positive traits that facilitate certain students to flourish academically in the face of challenging and unfamiliar circumstances [7,8,9]. Academic buoyancy and adaptability are two constructs that center on harnessing individuals’ strengths and emphasizing proactive rather than reactive approaches in dealing with academic setbacks, challenges, and changes [10,11]. The previously mentioned study utilizes the same dataset to explore how perceived parental involvement affects students’ academic buoyancy and adaptability, mediated by goal orientations [12]. As suggested by the implicit theories model, the diverse patterns of coping responses can also be shaped by the different beliefs about the nature of intelligence [13]. In light of this, our current manuscript builds upon this foundation by introducing a new variable—growth mindset—examined through the lens of implicit theories. This shift in focus addresses a distinct aspect of academic psychology by exploring how a growth mindset influences academic buoyancy and adaptability, and subsequently, academic achievement.

We differentiate the current study from our earlier work by elaborating on cognitive-behavioral and affective adaptability as specific dimensions of adaptability, which were not distinctly analyzed in the previous study. This allows us to present nuanced insights into how different facets of adaptability interact with academic buoyancy and a growth mindset to influence academic achievement, particularly in mathematics.

The scientific contribution of the current study lies in its comprehensive examination of the mediating roles of academic buoyancy and both forms of adaptability in the relationship between a growth mindset and academic performance. This extends the theoretical framework of implicit theories within the domain of positive psychology and provides empirical support for targeted interventions aimed at fostering a growth mindset to enhance educational outcomes.

Academic buoyancy refers to the ability to deal with low to moderate academic adversities that the vast majority of students may face in their daily school lives [10,14]. The examples of such challenges encompass the pressures of examinations, difficult homework, receiving grades lower than anticipated, facing urgent deadlines, experiencing declines in motivation, and so forth. Becoming buoyant can help prevent minor yet subjectively significant challenges from escalating into major problems [15]. While buoyancy focuses on how individuals respond to adversity, adaptability refers to the ability to make appropriate cognitive, behavioral as well as affective adjustments needed to interact with new, uncertain, and/or changing situations, circumstances, and conditions [16,17]. For instance, in the school environment, students encounter the task of learning new material during their lessons and are required to take tests that include questions that they have not encountered before. In addition, they may experience changes in teachers and classmates, progressing from one stage of schooling to the next, and even transferring to different schools. Adaptability is positively related to class participation, the enjoyment of school, and positive academic intentions, and negatively related to self-handicapping and disengagement [17]. Given that academic adversities and changes are specific to different domains, it is crucial to investigate the role of academic buoyancy and adaptability within specific academic contexts.

Mathematics is a key subject in preparing for future employment [18], and a lot of effort has been made and interventions have been designed to improve students’ mathematics achievement. With the prevalence of the Programme for International Student Assessment (PISA) results, many researchers have shown great interest in determining the reason for the high levels of the mathematics performance of students in Confucian Heritage Cultures (CHCs) [19]. Some of these researchers have argued that such educational success can be attributed to specific cultural values, such as beliefs about the role of effort and hard work in academic performance [20,21]. However, there is little empirical research to support these ideas. Therefore, this research aims to offer insights into the implicit beliefs and adaptive psychological attributes related to mathematics learning in the context of CHCs.

## 2. Literature Review

This section presents the previous studies involving the relationship between a growth mindset and mathematics achievement, the potential mediating effects of academic buoyancy and adaptability, the pertinent Chinese cultural contexts, and the influence of covariates.

### 2.1. The Association Between a Growth Mindset and Mathematics Achievement

The association between a growth mindset and students’ academic achievement has been widely examined by a number of educational empirical studies. Tarbetsky et al. [3] explored the association between implicit theories and achievement among indigenous Australian students from 20 high schools and found that students who believed that intelligence is malleable tended to perform better in overall achievement than those who believed that intelligence is stable. They also confirmed that a growth mindset was positively associated with mathematics achievement in the domain-specific (mathematics) condition. Blackwell et al. [2] conducted a longitudinal study focusing on students who made the transition to junior high school and demonstrated that the students’ incremental view about intelligence was a positive predictor of their mathematics performance after controlling for prior achievement. Although similar conclusions about the relationship between a growth mindset and mathematics achievement have been drawn in other research [22,23], the underlying mechanism still needs further research. Given the “powerful impact of growth mindset messages upon students’ attainment” [24], mindset intervention programs have been used to improve students’ academic achievement. However, an intervention involving only a mindset is not always effective, and it is better to combine it with other interventions to increase its usefulness [25]. Therefore, more research is needed to enable a better understanding of the mediators of the relationship between a growth mindset and mathematics achievement.

Learners with both academic buoyancy and adaptability can regulate and protect themselves from the negative and novel traits that arise in a more failure-prone learning environment [26,27]. Due to the complexity of the concepts, formulas, and theorems in mathematics, negative attitudes towards mathematics are common for learners worldwide [28]. Although Chinese students have demonstrated a high performance in mathematics on various international assessments, there is still a concerning trend of declining interest and confidence in learning mathematics as they progress to higher grades [29,30]. A great deal of effort has been made to relieve mathematics anxiety and avoidance, but it may be more productive to build positive beliefs and behaviors before the appearance of such negative phenomena [31]. Previous research has shown that a growth mindset [32], academic buoyancy [33], and adaptability [34] enable mathematics learners to perform better. People with a growth mindset believe that they can “do mathematics” and know how to gain support, make an effort, and adopt good strategies in response to academic setbacks or high levels of demands [31]. Furthermore, considering that mathematics serves as a crucial basis for numerous career prospects and plays a vital role in global development as a cognitive skill [35], this research aims to offer insights into the functioning of a growth mindset, academic buoyancy, and adaptability in the realm of mathematics learning.

### 2.2. Growth Mindset as an Antecedent of Academic Buoyancy and Adaptability

When faced with academic challenges, students’ implicit theories about intelligence can determine their responses [1,5,36]. Zhao et al. [37] measured the relationship between a growth mindset and grit and found that a growth mindset tended to foster more self-directed and autonomous learning motivation among students, which increased their overall grit. When confronting the persistent threat of failure, students who possess a growth mindset are more inclined to adopt remedial strategies and exhibit mastery-oriented patterns in comparison to those who possess a fixed mindset [38]. In particular, Doron et al. [39] explored the role of students’ implicit theories in determining the strategies which students used to cope with examinations. They found that when students approached an examination, a growth mindset was positively related to adaptive strategies, such as active coping, acceptance, searching for social assistance, and relieving negative emotions in order to deal with examinations, whereas a fixed mindset tended to be related to a disengagement from exams, which resulted in the avoidance of acceptance and active coping.

A noteworthy parallel to the role of implicit theories in the related domain of academic buoyancy can also be found. When students hold the belief that their intelligence is changeable, they perceive that working hard, rather than a lack of ability, is key to academic success and in turn respond buoyantly when faced with adversity [36,40]. In a sample of 504 Philippine high school students, Valdez [41] found that a growth mindset was strongly and positively correlated with academic buoyancy. In addition, as Martin et al. [17] suggested, in line with the growth perspective on the academic process, adaptability should be included in growth-related conceptual and applied frameworks. Adaptability is positively related to a growth mindset but negatively related to a fixed one [42]. The failure attribution may explain the reasons why an intelligence mindset can affect academic buoyancy and adaptability. Different mindsets lead students to adopt distinct attributions in situations involving challenges and setbacks [43,44]. Individuals who possess a growth mindset tend to attribute their failures to factors that are more within their control, such as strategy or effort [45]. Consequently, they are inclined to exert greater effort to enhance their performance in challenging or novel situations. The above mindset-related consequences can contribute to the development of academic buoyancy and adaptability. Taken together, therefore, students with a growth intelligence mindset may have higher levels of academic buoyancy and adaptability. However, to the best of our knowledge, little is known about how this joint construct is associated with a growth mindset and its unique role in the subject of mathematics.

### 2.3. Effects of Academic Buoyancy and Adaptability on Mathematics Achievement

Many constructs related to the responses during challenging conditions, such as grit and coping, have generally been found to have an impact on mathematics performance. He et al. [46] conducted a study involving a sample of 2931 seventh-grade students in Northwest China. The study revealed a significant and positive association between grit and mathematics achievement among the average students. Moreover, instead of treating grit as a unidimensional construct, Duckworth et al. [47] have proposed a two-dimensional construct of grit: perseverance of effort and consistency of interest. Building upon this framework, Guo [48] investigated 926 secondary school students with a specific focus on mathematics-related grit and its impact on the learning outcomes. The findings demonstrated that both perseverance of effort and consistency of interest exhibited a direct positive relationship with mathematics performance. Notably, the effect of perseverance of effort was found to be stronger compared to that of consistency of interest. In addition, the use of appropriate coping mechanisms is considered one of the plausible factors contributing to success in mathematics. A task-oriented coping was found to be positively associated with mathematics achievement [49,50].

Similar to grit and coping, the ability to respond to daily learning challenges and changes is critical to students’ academic success. There is a notable trend to extend previous subject-general research on academic buoyancy and adaptability to subject-specific investigations [33]. In particular, the relation of academic buoyancy and adaptability to mathematics achievement has received much attention. For instance, Martin and Marsh [10] identified buoyancy in the mathematics domain and found that the endorsement of academic buoyancy was positively associated with students’ academic performance. Weißenfels et al. [9] recognized the necessity of considering mathematics in the face of setbacks at school, given the diminishing students’ value attributed to the subject and the rising levels of mathematics anxiety. They further validated the positive association between academic buoyancy and mathematics achievement through an analysis of 974 secondary school students. Collie and Martin [34] used 371 students in grades 7–9 and their teachers as the target population to examine the impact of mathematics adaptability on academic scores. The findings revealed that both kinds of adaptability reported by students and teachers showed a positive impact on students’ mathematics performance. Although these studies examined the effect of both academic buoyancy and adaptability on mathematics learning, most research has examined these two constructs separately, and little is known about their joint effects.

Taken together, the existing literature supports the proposition that academic buoyancy and adaptability are associated with higher mathematics achievement. At the same time, as we elaborated earlier, implicit theories of intelligence are prone to relate strongly to mathematics achievement, academic buoyancy, and adaptability. Consequently, we can assume that academic buoyancy and adaptability can function as mediators of the relationship between a growth mindset and mathematics achievement.

### 2.4. The Chinese Culture

The effect of a student’s mindset and learning ability may depend on the learning context. In the Chinese cultural context, great emphasis is placed on education, and regarding education as the path to satisfying the desire for personal growth, knowledge expansion, and filial piety is one of the moral elements of Confucianism. Most people believe that academic accomplishments have a strong relationship with an individual’s social status, and that attaining high academic performance can bring honor to family members and repay parents’ sacrifice and investment [51]. This has resulted in highly competitive and exam-oriented school environments [52]. From a young age, Chinese children are educated to be persistent, concentrated, self-disciplined, and diligent in academic matters [53], all of which shape their mindset in that they believe more in effort and less in innate intelligence. This mindset promotes children’s ability to deal with the difficulties and challenges that they face in their daily school lives. In contrast, children in Western countries tend to perceive that innate ability, rather than effort, is a critical contributor to educational outcomes [21], and they may consider certain motivational orientations to be maladaptive [54]. Such differences in Eastern and Western children’s conceptions show that a growth mindset, academic buoyancy, and adaptability in China may be more obvious than in Western contexts.

Despite the prominent role of a growth mindset, buoyancy, and adaptability in academic learning in the Chinese culture, little empirical research has examined these constructs in the Chinese school setting. Thus, this research aims to provide some information that may contribute to a better understanding of this mindset and the ability-related structures underpinning the learning of Chinese students.

### 2.5. Relevant Covariates

In analyzing the relationship between a growth mindset, academic buoyancy and adaptability, and mathematics achievement, it is essential to control for potentially confounding factors in obtaining the unique variance. Previous studies suggest that family socioeconomic status (SES), gender, and school type may be related to these four study variables in the current study. In terms of family SES, researchers have found that a growth mindset is positively linked to learning only among higher SES students and not among lower-SES students [55,56]. Besides, higher family SES was found to be correlated with higher academic buoyancy and adaptability [57]. Children with lower SES tend to be more susceptible to stress, whereas those with higher family SES seem to benefit from protective factors when exposed to learning difficulties [58]. Moreover, the effects of family SES on academic achievement have been widely found [59]. High-SES students tend to have more parental involvement and more potential to obtain family learning resources, so they achieve higher academic performance than their-low SES peers [60]. Thus, family SES was included as one covariate in this study.

In terms of gender-related effects, researchers have also found that gender may have an impact on the four constructs. Females tend to attribute their failure to their lack of ability and, compared with their male counterparts, are less likely to seek help when facing learning challenges [61], and tend to underestimate their ability wrongly [62]. In addition, the differences in mathematics learning between boys and girls have been widely acknowledged [63]. Influenced by gender stereotypes, most girls believe that they do not have as much talent in learning mathematics as boys, which results in differences in mathematics performance [64]. Therefore, gender was another covariate in this study.

In terms of school-type effects, boarding students reside on campus throughout the academic term, returning home only during school breaks. In contrast, day students commute daily from home to school and return home after classes. Boarding students, due to their immersive school environment, are often afforded greater independence, self-discipline, and maturity [65,66]. They are responsible for managing their time, social interactions, and personal affairs within the school setting. In addition, the boarding school environment fosters closer relationships with both teachers and peers, promoting higher levels of teacher and peer support, although it may result in less parental involvement in daily school activities [67,68]. Researchers have identified that day or boarding school attendance experiences have the potential to shape the development of students’ intelligence mindsets [69]. Day and boarding students exhibited different levels of academic buoyancy and adaptability [70]. Furthermore, the implementation of study routines and schedules in boarding schools has been found to have a positive impact on the mathematics achievement of secondary school students [71]. Therefore, to account for the potential effect of school type, it was considered as a covariate in this study.

### 2.6. The Present Study

**Figure 1:**
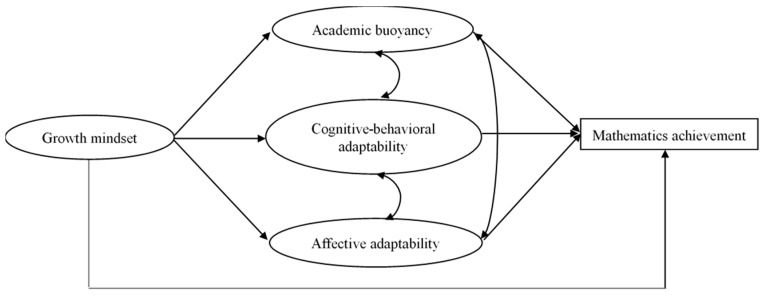
Hypothesized model of the relationship between growth mindset and mathematics achievement.

In sum, the present research conceptualizes the relationship between a growth mindset, academic buoyancy, adaptability, and mathematics achievement (see Figure 1). Drawing on the implicit theories model [5] and previous research, we posit that a student intelligence mindset is associated with student academic achievement. Further, student academic buoyancy and adaptability mediate this relationship. In particular, in contributing to a better understanding of adaptability, this study enriches previous studies by differentiating adaptability into cognitive-behavioral and affective adaptability and exploring the separate roles played by these sub-constructs in the hypothesized model. To examine whether the relationship between a growth mindset, academic buoyancy, adaptability, and mathematics achievement differs based on the demographic factors drawn from covariates, this study compares the invariance across the genders, school types, and SES levels. To be specific, the following four research questions will be addressed in the present research:

RQ1. Is a growth mindset related to mathematics achievement?

RQ2. Is a growth mindset related to academic buoyancy, cognitive-behavioral, and affective adaptability?

RQ3. Does academic buoyancy, cognitive-behavioral and affective adaptability mediate the association between a growth mindset and mathematics achievement?

RQ4. Does the relationship between a growth mindset, academic buoyancy, cognitive-behavioral adaptability, affective adaptability, and mathematics achievement vary across the genders, school types, and SES levels?

## 3. Method

### 3.1. Participants and Procedure

The survey took place in Guangzhou, a Tier 1 city in China. The researchers personally delivered invitation letters to the principals of ten secondary schools. Finally, seven schools expressed their willingness to collaborate with the research, comprising three boarding schools and four day schools. Within each school, four to eight 8th-grade classes were randomly selected, and all the students and their parents from the selected classes were invited to participate in this study. Both the students and parents received consent forms informing them about the purpose of the research. When the students’ and parents’/guardians’ consent had been obtained, the students were asked to complete a 40 min mathematics assessment and then a 20 min questionnaire in a paper-and-pencil format in their classrooms. The students completed the mathematics assessment under normal examination conditions. The parent questionnaire evaluating the family SES, which would take approximately 10 min to complete, was taken home by the students for their parents to complete. A total of 1295 students from 38 classes and their parents were provided with these materials. However, 131 students did not return the tests or questionnaires. Therefore, the final dataset comprised 1164 students, reflecting a response rate of 89.9%, who completed and returned all the necessary materials. This dataset provided reliable and valid information for the subsequent data analysis. Among the participants, 589 (50.6%) were boys, while 575 (49.4%) were girls.

### 3.2. Measures

#### 3.2.1. Growth Mindset

The Implicit Theories of Intelligence Scale for Children [1] was used to assess the students’ growth mindset. The original scale consists of six items: three fixed mindset statements (e.g., ‘‘You have a certain amount of intelligence, and you really can’t do much to change it’’) and three growth mindset statements (e.g., ‘‘You can always greatly change how intelligent you are’’). However, this study only used the three fixed mindset statements. Some researchers have found that when participants are offered the options of both mindsets, they seem unable to resist the too-compelling statements concerning a growth mindset, even for those with fixed mindsets [72,73]. Furthermore, the combination of growth and fixed mindset items may lead to confusion among students due to the repetition of the same idea [1]. Research has demonstrated that disagreement with fixed mindset statements can signify agreement with a growth mindset [5,72]. Thus, in order to mitigate the impact of social desirability and repetition effects, it is appropriate to use the fixed mindset choices only. The participants rated the items from 1 (strongly disagree) to 7 (strongly agree). The items were reverse scored, and the higher the subscale scores, the more a growth mindset the students had. This approach to measuring a growth mindset has been utilized in earlier research and has consistently demonstrated high internal reliability [5,74,75,76,77]. Within the current study, this three-item scale also had good internal consistency (Cronbach’s alpha = 0.895, 0.884, and 0.907 for the full sample, boys, and girls).

#### 3.2.2. Academic Buoyancy

To assess the students’ academic buoyancy in mathematics learning, a four-item scale was developed based on the Academic Buoyancy Scale (ABS) [10]. Some adjustments were made to highlight the features of mathematics (e.g., “I don’t let the stress of studying mathematics get on top of me.”). The participants rated the items ranging from 1 (strongly disagree) to 7 (strongly agree). A higher score indicated a higher level of academic buoyancy. This scale was internally consistent (Cronbach’s alpha = 0.868, 0.871, and 0.854 for the full sample, boys, and girls).

#### 3.2.3. Adaptability

We developed a nine-item scale of students’ adaptability in mathematics learning based on the Adaptability Scale [17,42]. All the items were adjusted to concentrate on mathematics learning. The first six items concerned the “cognitive-behavioral” factor (e.g., “While learning mathematics, I am able to think through a number of possible options to assist me in a new situation.”). The remaining three items concerned the “affective” factor (e.g., “While learning mathematics, I am able to reduce negative emotions [e.g., fear] to help me deal with uncertain situations.”). The participants rated the items from 1 (strongly disagree) to 7 (strongly agree). A higher score indicated a higher level of cognitive-behavioral or affective adaptability. Both subscales demonstrated strong internal consistency. Specifically, for cognitive-behavioral adaptability, the Cronbach’s alpha coefficients were 0.921, 0.928, and 0.910 for the full sample, boys, and girls, respectively. As for affective adaptability, the Cronbach’s alpha coefficients were 0.884, 0.870, and 0.888 for the full sample, boys, and girls, respectively.

#### 3.2.4. Mathematics Achievement

The mathematics test used in the current study was adapted from the one used by Chen et al. [78]. Chen et al. developed the test based on the released items (8th grade) from the Trends in International Mathematics and Science Study (TIMSS). This test is composed of twenty-nine items (some items include a set of sub-questions, and thus there are thirty-seven sub-items in total), and its maximum score is 46. There are two item format types—selected-response (multiple-choice) and constructed-response items—covering four domains of content (i.e., geometry, number, data and probability, and algebra) and three domains of cognition (i.e., knowing, applying, and reasoning). In previous research, this test has been used to examine Chinese students, and all the items performed well in difficulty and discrimination. After obtaining permission from the authors, we used this test as the first version of the mathematics test. Then, we invited five experienced mathematics teachers to review the test to check the appropriateness of the content and cognitive domain, and the accuracy of the translation. Some teachers found that item 17 and item 25 covered the same content and cognitive domain and had the same type of questions. After discussion, we reached an agreement on the deletion of item 25 and finalized the test with the remaining twenty-eight items (thirty-six sub-items, with a maximum total score of 44).

A psychometric analysis of the thirty-six sub-items with the 1,164 students was conducted. The results, calculated by using the formula of classic test theory, indicated that the difficulty and discrimination parameters were good. The indices of item difficulty differed from 0.09 to 0.93 (M = 0.69, SD = 0.19), and the indices of item discrimination (the Upper-Lower Difference Index) varied from 0.12 to 0.71 (M = 0.44, SD = 0.17). As for the difficulty parameters, one item was difficult (*p* < 30%), fifteen items were within the recommended range (*p* = 30–70%), and the remaining twenty items were relatively easy (*p* > 70%) [79]. Of the thirty-six sub-items, four had poor discrimination parameters (<0.20), six exhibited acceptable results, ranging from 0.20 to 0.29, four were within a good range (0.30–0.39), and the remaining twenty-two items showed excellent discrimination parameters (>0.39) [80]. In the subsequent analysis, the raw scores were converted into standardized z-scores.

#### 3.2.5. Student Gender, School Type, and Family SES

We included student gender (0 = female; 1 = male), school type (0 = day student; 1 = boarding student), and students’ family SES in the investigation. A student’s parents or guardians reported three indicators of family SES: parental education, parental occupation, and family learning resources. For parental education, the participants were asked to provide information about their educational level (e.g., 0 = no education, 1 = elementary school, 2 = junior high school, and 3 = high school). For parental occupation, the participants were required to report their occupation and job category. Based on the Chinese Occupational Prestige Measuring Index (COPMI) [81], occupations and jobs were categorized from the highest to the lowest level, with a score ranging from 7 to 1. The maximum of the mother’s and the father’s educational (or occupational) score was viewed as the score of the parents’ education (or occupation). For family learning resources, the participants were asked to report the availability of resources such as a desk at home to study at, a quiet place to study, and online mathematics course resources. They were also asked to report the number of books, televisions, cell phones with Internet access, computers, tablet computers, and e-book readers there were in the home environment. After converting the raw sums into standardized z-scores, we obtained a summary index of the family learning resources. Finally, an aggregate score of family SES was determined after entering the parental education, parental occupation, and family learning resources into factor analysis via a principal component method of extraction. A single factor showing 57.2% of the variance across the three variables was extracted. Although 57.2% was slightly low, it is common and acceptable in social sciences [82,83,84]. A higher score represented a higher SES. This calculation method of family SES has been used before [85,86,87].

### 3.3. Data Analyses

In the hypothesized model, the independent variable was a growth mindset. The mediating variables were academic buoyancy, cognitive-behavioral adaptability, and affective adaptability. The dependent variable was mathematics achievement. Student gender, school type, and family SES were three controlled variables. The present study adhered to the recommended criteria for the participant-to-item ratio (10:1) [88], as the sample size chosen was sufficient to accommodate the total number of items across three scales and the mathematics test, which amounted to 44. In the sample of 1164 students, SPSS 21.0 was used to conduct the descriptive and Pearson correlation analyses of all the variables, with pairwise deletion employed to account for missing data. Mplus 7.4, with the default maximum likelihood (ML) estimator, was used to test the models. The full-information maximum likelihood (FIML) method was used to handle missing data [89].

To assess the common method bias, the Harman single factor test was employed [90]. Then, before the structural model was run to examine the hypotheses, confirmatory factor analysis (CFA) was conducted to confirm the factorial structures of the Implicit Theories of Intelligence Scale, the Academic Buoyancy Scale, and the Adaptability Scale [91]. After that, structural equation modeling (SEM) was applied to assess the mediation model. The indirect effects were tested via bootstrap analyses considering 95% confidence intervals (1000 iterations) [92]. The confidence intervals of all the significant indirect paths did not contain zero. A model was considered an adequate fit based on the following indices: the root mean square error of approximation (RMSEA) and standardized root mean square residual (SRMR) which should be lower than 0.08; and the Tucker–Lewis Index (TLI) and the comparative fit index (CFI) which should be higher than 0.90 [93]. All the loading values of the items on the latent factors should not be lower than 0.4.

To examine the relationship between a growth mindset, academic buoyancy, adaptability, and mathematics achievement across the genders, school types, and SES levels, the multi-group SEM was used in the research. In particular, the SES scores were divided into three groups to sort the students: lower SES (students with scores in the first tertile), those with central SES (students with scores in the second tertile), and higher SES (students with scores in the third tertile). Prior to evaluating the structural invariance, it is necessary to investigate measurement invariance. In accordance with Vandenberg and Lance [94], the multi-group analysis should include testing for configural, metric, and scalar measurement invariance to assess its suitability across the genders, school types, and SES. In configural invariance, factor loadings and thresholds are unconstrained and allowed to vary across groups. Once configural invariance is established, the subsequent step involves examining metric invariance. In metric invariance, the factor loadings are constrained to be equal across groups. Following the confirmation of metric invariance, the analysis proceeds to assess the scalar invariance. In scalar invariance, the factor loadings and intercepts of all items are constrained to be equal across groups. Measurement invariance is considered to be established when two conditions are met: first, the overall model fit should be acceptable [95]; second, the change in the RMSEA (ΔRMSEA) should be less than or equal to 0.015, and the change in the CFI (ΔCFI) should be equal to or greater than −0.01 [96].

Following the establishment of the measurement invariance, an evaluation was conducted to determine the equivalence of the structural paths across the groups. Only the significant paths in the model were compared. In the analysis, we conducted a comparison between an unconstrained model, which allowed the structural paths to be estimated freely across the groups, and a constrained model, where the corresponding structural paths were fixed to be the same across the groups. To assess the change in model fit, a chi-square difference test was performed on the nested models, where a significant Δχ^2^(Δdf) value would indicate a significant change in the model fit [97]. When the chi-square test yielded significance, we proceeded to constrain the individual structural paths one at a time to determine if there were any significant differences; if no significance was found, the analysis was terminated.

## 4. Results

### 4.1. Common Method Bias

In testing for the common method bias, a single-factor CFA using all the items of the Implicit Theories of Intelligence Scale, the Academic Buoyancy Scale, and the Adaptability Scale revealed an extremely poor fit, with χ^2^ (104) = 4159.671, CFI = 0.708, TLI = 0.663, RMSEA = 0.183, and SRMR = 0.106. This indicated that the common method bias was unlikely to be a serious issue in this study.

### 4.2. Descriptive Statistics and Correlations

Table 1 and Table 2 present the descriptive statistics and Pearson correlations among the study variables within the entire sample, as well as separately within the male and female samples. In all three samples, a growth mindset, academic buoyancy, cognitive-behavioral adaptability, affective adaptability, and mathematics achievement were correlated with each other. The results of the full sample demonstrated that gender was positively associated with academic buoyancy, cognitive-behavioral adaptability, and affective adaptability, but not significantly correlated with a growth mindset and mathematics achievement. At the same time, the school type and family SES were positively related to a growth mindset, academic buoyancy, cognitive-behavioral adaptability, and mathematics achievement, but no significant relationship was found between the school type and affective adaptability, as well as between the family SES and affective adaptability. However, in the male and female samples, the correlation results for the school type and family SES show variations.

### 4.3. Testing the Measurement Model

In testing the measurement model with all the latent constructs related to their observed indicators, the CFA results showed that χ^2^ (98) = 758.873, CFI = 0.952, TLI = 0.942, RMSEA = 0.076, and SRMR = 0.038, indicating a good model fit for the three scales. Additionally, the standardized factor loadings of each indicator on the corresponding latent construct were all above 0.4 and significant at the *p* < 0.001 level, suggesting that the observed indicators actually represented their respective latent variables. Table 3 provides a summary of the survey items and CFA loadings for the scales, both for the full sample and separately for boys and girls.

### 4.4. Examining the Structural Model

**Figure 2:**
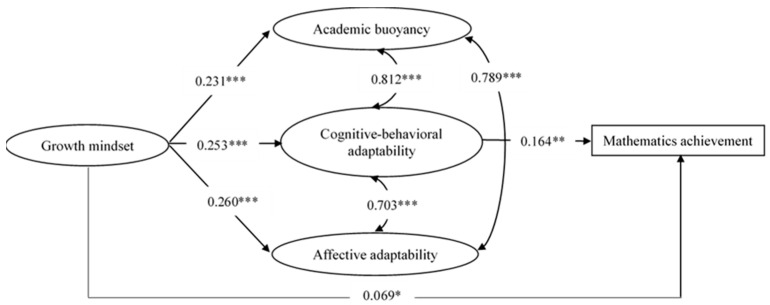
Structural equation model testing the relationship between growth mindset, academic buoyancy, cognitive-behavioral adaptability, affective adaptability, and mathematics achievement. Note. To simplify the view, we do not show the observed indicators of each latent variable in the figure. All the correlations and path coefficients shown in the figure are standardized and statistically significant (* *p* < 0.05, ** *p* < 0.01, *** *p* < 0.001).

The SEM results are presented in Figure 2 and demonstrate an acceptable model fit: χ^2^ (186) = 1052.943, CFI = 0.942, TLI = 0.928, RMSEA = 0.063, and SRMR = 0.037. The total R squares for mathematics achievement was 0.334. Table 4 shows the standardized estimates and 95% confidence intervals of both the direct and indirect paths. The direct path between a growth mindset and mathematics achievement was significant (β = 0.069, *p* < 0.05). The mediation effect of cognitive-behavioral adaptability in the relationship between a growth mindset and mathematics achievement was 0.042 (*p* < 0.05), indicating that cognitive-behavioral adaptability positively and partially mediated the correlation between a growth mindset and mathematics achievement. The mediation effect of academic buoyancy and affective adaptability in the relation between a growth mindset and mathematics achievement was not significant (β = 0.014 and −0.022, *p* > 0.05), indicating that academic buoyancy and affective adaptability did not mediate the relation between a growth mindset and mathematics achievement. A growth mindset explained 10.3% of the variance of mathematics achievement. The indirect effect of cognitive-behavioral adaptability accounted for 40.8% of the total effect of a growth mindset.

### 4.5. Multi-Group Analysis Across the Genders, School Types, and SES Levels

The measurement invariance test was conducted to assess whether the evaluations of the relevant variables by the participants were equivalent across the genders, school types, and SES levels. The results of the measurement invariance test showed that the overall model fits were acceptable, and ΔRMSEAs and ΔCFIs fell within the generally recommended criteria (Table 5), demonstrating that the proposed model attained configural, metric, and scalar invariances across the genders, school types, and SES tertiles. Thus, the analysis proceeded to examine the structural invariance.

In the evaluation of the structural invariance, the results of the chi-square difference test across the genders (Δχ^2^ = 3.468, Δdf = 5, *p* = 0.628), across the school types (Δχ^2^ = 4.9, Δdf = 5, *p* = 0.428), and across the SES tertiles (Δχ^2^ = 13.759, Δdf = 10, *p* = 0.184) showed that the overall fit for the constrained models did not significantly differ from the unconstrained models. This suggests that there were no significant differences observed in the path coefficients between the groups, meaning that the corresponding paths were equal for boys and girls, for boarding and day students, as well as for low-, middle-, and high-SES students.

## 5. Discussion

The findings of this study showed that a student growth mindset was positively related to mathematics achievement. Furthermore, a student growth mindset was significantly associated with student self-reported academic buoyancy, cognitive-behavioral adaptability, and affective adaptability, but only cognitive-behavioral adaptability further mediated the relationship between a student growth mindset and mathematics achievement. Extending the prior studies that have observed a positive relationship between a student growth mindset and learning outcomes mediated by students’ psychological or behavioral mediators, this study obtained evidence to support this relationship, based on students’ achievement-related abilities, and examined the possible mediating role of students’ academic buoyancy and adaptability in the relationship. The study also generalized the related constructs beyond the mainstream student populations. Research that investigates a mechanism underlying mathematics achievement may contribute to a better understanding of students’ broader approaches to mathematics learning and lay a foundation for more integrative educational interventions.

### 5.1. Growth Mindset and Mathematics Achievement

The present study found a positive correlation between a growth mindset and mathematics achievement, in line with the previous literature [22,23]. Researchers have discussed the distinctive role of students’ mindsets in determining their academic outcomes. One explanation for why students who reported a growth mindset had high academic performance may relate to their attitudes towards failure. Unlike entity beliefs, incremental beliefs offer more hope for personal improvement [13], which prevents students from viewing failure as a long-term threat to their intelligence and thus benefits their achievement. Another possible explanation is related to students’ achievement motivation. Promoting a growth mindset in students may help them become better motivated. Students’ beliefs sometimes act as a mental stimulus that students bring to the achievement situation [2]. In this respect, this phenomenon is more prominent among students from Confucian heritage regions because of their strong drive to achieve, their emphasis on effort and willpower, and a tendency for Chinese people to be less forgiving with respect to underachievement [98]. As students’ incremental theory of intelligence develops, it may begin to form a constellation with students’ beliefs about effort, attributions, and goals for achievement, resulting in these constructs connecting in a meaning system of beliefs and goals that have a real influence on boosting mathematics achievement.

### 5.2. Growth Mindset and Academic Buoyancy and Adaptability

Compared with most previous research, which investigated academic buoyancy and adaptability separately and used a unidimensional measurement of adaptability, the present research, by examining academic buoyancy and adaptability simultaneously, was able to explore the two constructs in greater detail. The positive association between a growth mindset and academic buoyancy and adaptability indicated that when students endorsed the belief that their intelligence was changeable, they were more likely to conquer learning difficulties, challenges, uncertainty, and novelty in their mathematics learning. These findings are in line with the literature on the role of students’ implicit self-theories in their academic-related thoughts, feelings, and behaviors [38,99]. The Confucian culture supplies the background in which a growth mindset operates in Chinese society. To be specific, influenced by the cultural values of Confucianism, when Chinese students hold a malleable and incremental view of intelligence and competence, their self-regulatory process and perseverance will be promoted, which underscores the positive impact of adversity and personal capacity on overcoming difficulties or any other kinds of complex situations [100].

Concerning the domain-specific (mathematics) condition, with the advent of complex tasks in mathematics curricula, a higher level of intelligence is required in problem solving [101], and a fixed mindset regarding mathematics learning and the idea that only a minority of people are “mathematics persons” are particularly prevalent [31]. Even so, mathematics has the potential to trigger distinctive motivational patterns related to a growth mindset, which manifest themselves in challenging situations [102]. At the same time, the mathematics learning process requires students to build upon previously learned knowledge, which may result in the accumulation of deficits in mathematics learning skills over time, thus producing longer-term differences in the trajectories of mathematics buoyancy and the two types of adaptability. Students who endorse a growth mindset tend to improve their level of intelligence by trying harder and making an effort. Such experiences are likely to support a positive view of dealing with challenges and changes in their mathematics learning.

### 5.3. The Mediating Role of Academic Buoyancy and Adaptability

This study found that cognitive-behavior adaptability significantly and partially mediates the relationship between a growth mindset and mathematics achievement, suggesting that implicit beliefs and cognitive and behavioral competence in response to changing, uncertain and new situations did play an important role in students’ mathematics outcomes. The mediating role of students’ cognitive-behavior adaptability in the present research is consistent with the implicit theories model discussed earlier. Unlike students with an entity mindset, who hold the belief that working hard is a display of incompetence, students with an incremental mindset believe that one should invest more effort into developing one’s intellectual ability, so they are more likely to be cognitively and behaviorally engaged in academic learning [73]. At the same time, students with a growth mindset are likely to feel a greater sense of autonomy and control over their academic success. Such a sense has been shown to positively predict the academic behaviors that help students learn more [103], such as help-seeking in different circumstances [104]. These components for gains in students’ mathematics achievement are especially important. For example, when learning mathematics, students usually need to deal with tasks that bring about uncertainty, like competing claims, open-ended problems, and non-readily verifiable outcomes [105]. Students with a growth mindset may believe that their ability in solving such tasks can be developed through hard work, and they are willing to invoke higher-order cognition and spend more time thinking and practicing, which finally leads to deep mathematical understanding and meaningful learning. Taken together, the direct and indirect path analyses reflect the importance of considering both students’ implicit theory of intelligence and their cognitive-behavioral adaptability when attempting to improve their levels of mathematics learning outcomes.

However, contrary to our hypothesis, neither academic buoyancy nor affective adaptability mediated the relation between a growth mindset and mathematics achievement, which was inconsistent with earlier research examining the effect of students’ beliefs about intelligence on academic performance via achievement-related reactions in complex learning situations [106,107]. This phenomenon might occur because academic buoyancy and affective adaptability, which are correlated with a growth mindset, may make students concentrate more on long-term learning than on short-term academic outcomes [108], which will lead to temporary fluctuations in mathematics achievement. In future investigations, the roles of academic buoyancy and affective adaptability in the association between a growth mindset and mathematics achievement can be further explored.

### 5.4. Covariates and Core Variables

Gender, school type, and family SES were included primarily as covariates to control for the variance in the current research, and there were informative associations between the core variables and the covariates that deserve discussion. First, as expected, the current study found that male students reported higher levels of academic buoyancy and the two types of adaptability, which was consistent with previous research [10,109,110,111]. Females experience higher levels of mathematics anxiety than males [112], and they report greater stress from academic hassles and are more susceptible to the negative impact of ongoing daily stressors in their learning environment [113]. This may lead female students to adopt the maladaptive pattern of coping in the face of challenges or some other situations that they have never met. These gender effects on the relevant constructs lead to the different correlation results of another two controlled variables (i.e., school type and family SES) within the male and female samples.

Second, the results in the full sample showed that boarding or high-SES students were more inclined to view their intelligence as changeable, had higher abilities to deal with everyday learning adversities, and were able to make appropriate cognitive and behavioral adjustments to interact with new, uncertain, and/or changing situations. These were partially consistent with prior research [57,69,114,115]. Boarding students are more prone to being exposed to a broader range of social, cultural, and educational capital within the school setting, and boarding schools typically implement highly structured study routines and schedules, fostering an environment conducive to focused and disciplined study habits [116]. Students from higher-SES families were also more motivated to study [117] and exhibited higher achievement-striving standards [118]. Third, in line with previous research [119,120,121], boarders or high-SES students demonstrated superior performance in mathematics compared to their peers.

In the multi-group analysis, no significant differences in the path coefficients across the subgroups were found, implying that while the variations in the levels of growth mindset, academic buoyancy, adaptability, and mathematics achievement may exist among different student demographics, the associations between these variables appear to be relatively similar across the genders, school types, and SES levels. As such, future efforts aimed at enhancing a growth mindset, academic buoyancy, and adaptability may lead to comparable effects on mathematics learning outcomes irrespective of the gender, school type, and family SES.

## 6. Limitations and Future Research Directions

A few limitations of this study need to be addressed in future studies. First, all the constructs in the current research were assessed by way of self-report measurements. Although we assume that self-report data reflect the participants’ real behaviors and thoughts, it is possible that what participants report is not consistent with their actions. For example, sometimes people report a growth mindset in the questionnaire survey but act in another way [122]. Future research can use various assessments, apart from Likert-style surveys, such as interviews or observations for the triangulation of the scale-based responses. Second, a domain-general scale was used to measure the students’ growth mindset. The high reliability of the scale in the present study indicated that such domain-general measurement is in line with the underlying construct in the area of mathematics learning, but future work can consider adjusting the items to concentrate on mathematics-specific measures of a growth mindset in order to provide a deeper explanation of how growth constructs function in mathematics. Third, given that the selection of schools and classes for investigation relied on convenience sampling and the willingness of school principals, sampling weights were not incorporated for the statistical analysis. However, it is important to acknowledge that the exclusion of sampling weights could potentially constrain the generalizability of the findings. Future research can employ more representative and diverse samples and incorporate appropriate sampling weights in order to strengthen the robustness of the findings and enhance the validity of the results. Lastly, this study employed a cross-sectional design, which assessed the students’ growth mindset, academic buoyancy, adaptability, and mathematics achievement at a single time point and organized the order of constructs based on prior research and theories [5,6]. In future work, longitudinal research can be utilized to investigate the causal ordering of these constructs.

## 7. Conclusions and Implications

This is the first empirical study to combine growth mindset, academic buoyancy, and adaptability to indicate their contributions to student mathematics achievement. It expands the implicit theories model by identifying the mechanism underlying the effect of a growth mindset on student learning in a specific subject and reveals the role of culture in shaping both the beliefs about intelligence and the reactions in dealing with academic challenges and changes in the Chinese context.

The findings of the current study have ramifications for practical intervention. It is essential to realize that without considering students’ abilities and psychological problems in the face of increasingly complex situations, educational reform efforts to improve curricula and instruction may be less effective than hoped. On this note, this research indicates that when conducting mindset interventions for students, practical efforts to cultivate students’ cognitive-behavior adaptability may be a useful approach to reducing the achievement gap in a difficult subject such as mathematics. Thus, we suggest that school stakeholders should create a supportive school environment that emphasizes personal growth and adaptability, and make use of theory and practices from the existing knowledge to introduce the importance and development of students’ mindset and coping skills in teachers’ professional development programs and the parent–school collaboration works. Teachers can foster a learning climate focusing on incremental development, such as organizing intelligence training activities, alleviating students’ mental “baggage” and encouraging positive responses in dealing with situations that are full of challenges, novelty, or uncertainty in mathematics learning. Parents should transfer their focus from children’s mathematics performance per se to effective household communication and strategies to shape children’s healthy beliefs and attitudes about their intelligence, strengthen their positive cognition and behaviors, and provide targeted support during the mathematics learning process.

## Figures and Tables

**Table 1:** Descriptive and correlation results of study variables within the full sample (N = 1164).

	1	2	3	4	5	6	7	8
1 Gender	-							
2 School type	0.051	-						
3 Family SES	−0.023	−0.027	-					
4 Growth mindset	0.010	0.091 **	0.095 **	-				
5 Academic buoyancy	0.217 ***	0.063 *	0.100 ***	0.246 ***	-			
6 Cognitive-behavioral adaptability	0.184 ***	0.089 **	0.135 ***	0.277 ***	0.736 ***	-		
7 Affective adaptability	0.188 ***	0.057	0.054	0.280 ***	0.723 ***	0.698 ***	-	
8 Mathematics achievement	0.044	0.097 ***	0.414 ***	0.174 ***	0.208 ***	0.242 ***	0.144 ***	-
M	0.510	0.470	0.000	5.560	5.111	5.102	5.253	0.000
SD	0.500	0.500	1.000	1.420	1.348	1.174	1.329	0.999

Note. * *p* < 0.05, ** *p* < 0.01, *** *p* < 0.001.

**Table 2:** Descriptive and correlation results of study variables within boys and girls.

	1	2	3	4	5	6	7
**A—boys**							
1 School type	-						
2 Family SES	−0.065	-					
3 Growth mindset	0.087 *	0.111 **	-				
4 Academic buoyancy	0.060	0.177 ***	0.206 ***	-			
5 Cognitive-behavioral adaptability	0.086 *	0.171 ***	0.246 ***	0.749 ***	-		
6 Affective adaptability	0.054	0.114 **	0.253 ***	0.687 ***	0.691 ***	-	
7 Mathematics achievement	0.106 **	0.426 ***	0.203 ***	0.264 ***	0.282 ***	0.175 ***	-
M	0.500	−0.022	5.575	5.400	5.316	5.500	0.043
SD	0.500	0.962	1.428	1.333	1.192	1.248	0.988
**B—girls**							
1 School type	-						
2 Family SES	0.012	-					
3 Growth mindset	0.095 *	0.081	-				
4 Academic buoyancy	0.046	0.040	0.296 ***	-			
5 Cognitive-behavioral adaptability	0.076	0.112 **	0.319 ***	0.700 ***	-		
6 Affective adaptability	0.040	0.012	0.310 ***	0.736 ***	0.686 ***	-	
7 Mathematics achievement	0.084 *	0.407 ***	0.145 ***	0.141 ***	0.189 ***	0.103 *	-
M	0.450	0.0229	5.546	4.815	4.884	5.000	−0.044
SD	0.498	1.038	1.414	1.299	1.114	1.362	1.011

Note. * *p* < 0.05, ** *p* < 0.01, *** *p* < 0.001.

**Table 3:** Items and CFA loadings for the scales used in this study.

Item	Boys (N = 589)	Girls (N = 575)	Full Sample (N = 1164)
**Scale 1: Growth mindset**			
GM1	0.856 ***	0.931 ***	0.893 ***
GM2	0.983 ***	0.958 ***	0.969 ***
GM3	0.721 ***	0.748 ***	0.735 ***
**Scale 2: Academic buoyancy**			
AB1	0.730 ***	0.708 ***	0.722 ***
AB2	0.854 ***	0.841 ***	0.856 ***
AB3	0.748 ***	0.748 ***	0.754 ***
AB4	0.823 ***	0.791 ***	0.816 ***
**Scale 3: Adaptability**			
*Subscale: Cognitive-behavioral adaptability*			
CBA1	0.842 ***	0.866 ***	0.860 ***
CBA2	0.883 ***	0.914 ***	0.902 ***
CBA3	0.843 ***	0.756 ***	0.804 ***
CBA4	0.796 ***	0.698 ***	0.735 ***
CBA5	0.824 ***	0.765 ***	0.803 ***
CBA6	0.764 ***	0.716 ***	0.746 ***
*Subscale: Affective adaptability*			
AA1	0.854 ***	0.880 ***	0.873 ***
AA2	0.867 ***	0.904 ***	0.886 ***
AA3	0.777 ***	0.784 ***	0.787 ***

Note. GM growth mindset, AB academic buoyancy, CBA cognitive-behavioral adaptability, AA affective adaptability. *** *p* < 0.001.

**Table 4:** Standardized direct and indirect paths in SEM (N = 1164).

Paths	β	95%CI	
		Low	High
Growth mindset—Mathematics achievement	0.069 *	0.010	0.120
Growth mindset—Academic buoyancy—Mathematics achievement	0.014	−0.024	0.057
Growth mindset—Cognitive-behavioral adaptability—Mathematics achievement	0.042 *	0.011	0.080
Growth mindset—Affective adaptability—Mathematics achievement	−0.022	−0.056	0.009
Sum of effects	0.103 ***	0.043	0.156

Note. * *p* < 0.05, *** *p* < 0.001.

**Table 5:** Fit indices for measurement invariance tests of the model across (A) genders, (B) school types, and (C) SES levels.

Model	χ^2^	df	RMSEA (ΔRMSEA)	CFI (ΔCFI)	TLI	SRMR
**A—gender (N_boy_ = 589, N_girl_ = 575** **)**						
Configural invariance	1135.022	342	0.063	0.946	0.933	0.041
Metric invariance	1181.080	361	0.062 (−0.001)	0.944 (−0.002)	0.935	0.051
Scalar invariance	1351.764	380	0.066 (0.004)	0.934 (−0.01)	0.927	0.064
**B—school type** **(N_boarding_ = 552, N_day_ = 612)**						
Configural invariance	1229.784	342	0.067	0.941	0.927	0.041
Metric invariance	1254.523	361	0.065 (−0.002)	0.940 (−0.001)	0.931	0.047
Scalar invariance	1319.179	380	0.065 (0.000)	0.937 (−0.003)	0.931	0.054
**C—SES** **(N_lowest tertile_ = 388, N_middle tertile_ = 385, N_highest tertile_ = 391)**						
Configural invariance	1290.820	402	0.075	0.938	0.922	0.041
Metric invariance	1335.490	434	0.073 (−0.002)	0.937 (−0.001)	0.927	0.052
Scalar invariance	1365.931	466	0.071 (−0.002)	0.938 (0.001)	0.932	0.052

## Data Availability

The data are not publicly available due to privacy or ethical restrictions, but they will be available from the corresponding author upon reasonable request.
